# Predictors of survival in patients with sarcoma admitted to the intensive care unit

**DOI:** 10.1186/s13569-016-0051-5

**Published:** 2016-07-19

**Authors:** Rohan Gupta, Neda Heshami, Chouhan Jay, Naveen Ramesh, Juhee Song, Xiudong Lei, Erfe Jean Rose, Kristen Carter, Dejka M. Araujo, Robert S. Benjamin, Shreyaskumar Patel, Joseph L. Nates, Vinod Ravi

**Affiliations:** The University of Texas at Houston Internal Medicine Residency Program, Houston, TX USA; The University of Texas Graduate School of Biomedical Sciences at Houston, Houston, TX USA; Division of Quantitative Sciences, The University of Texas MD Anderson Cancer Center, Houston, TX USA; Department of Critical Care, The University of Texas MD Anderson Cancer Center, Houston, TX USA; Department of Sarcoma Medical Oncology, The University of Texas MD Anderson Cancer Center, 1515 Holcombe Blvd # 450, Houston, TX 77030 USA

**Keywords:** Cancer, Sarcoma, ICU, Survival, SOFA

## Abstract

**Background:**

Advances in treatment of sarcoma patients has prolonged survival but has led to increased disease- or treatment-related complications resulting in greater number of admissions to the intensive care unit (ICU). Survival and long-term outcome information about such critically ill patients with sarcoma is unknown.

**Methods:**

The primary objective of the study was to determine the ICU and post-ICU survival rates of critically ill sarcoma patients. Secondary objectives included determining the modifiable and non-modifiable predictors of poor survival. We performed a retrospective chart review of sarcoma patients admitted to the ICU at The University of Texas MD Anderson Cancer Center between January 1, 2005, and December 31, 2012. Main outcome measures were ICU mortality, in-hospital mortality and 1, 2, and 6-month survival rates. Covariates such as histological diagnosis, disease characteristics, chemotherapy use, Charlson comorbidity index, Sequential Organ Failure Assessment (SOFA) scores, and clinical findings leading to ICU admission were analyzed for their effects on survival.

**Results:**

We identified 172 admissions over the 8-year study period hat met our inclusion criteria. The study population was 45.9 % males with a median age of 52 years. The most common sarcoma subgroups were high-grade unclassified sarcoma (25 %) and bone tumors (17.4 %). The ICU mortality rate was 23.3 % (95 % confidence interval [CI], 16.9–29.6 %), and an additional 6.4 % of patients died before hospital discharge (95 % CI, 22.9–37.1 %). 6-month OS rates were 41 %. The median SOFA scores on admission were 6 (inter quartile range (IQR), 3.5–9) in ICU survivors and 10 (IQR, 6.5–14) in ICU non-survivors. Increase in SOFA scores ≥6 led to poor outcomes (ICU survival 13.3 %, OS 6.7 %). Charlson comorbidity index (HR 1.139, 95 % CI 1.023–1.268, p = 0.02) and discharge SOFA scores (HR 1.210, 95 % CI 1.141–1.283, p < 0.0001) correlated with overall survival.

**Conclusions:**

Our results suggest that patients that are admitted to the ICU for respiratory failure, cardiac arrest, septic shock, acute renal failure or acidosis and also have a high SOFA score with subsequent worsening in the ICU have poor prognosis. Based on the retrospective data which needs further validation we can recommend that judicious approach should be taken in patients with predictors of poor survival before subjecting them to aggressive treatment.

## Background

Sarcomas are a rare, histologically and behaviorally diverse group of malignant connective tissue tumors that make up approximately 1 % of all adult malignancies and 12 % of pediatric cancers [1, 2]. As a result of the substantial progress made in the past 2 decades in understanding the behavior and molecular pathogenesis of sarcoma, new therapies have been developed. Advances in treatment may prolong survival but can lead to increased disease- or treatment-related complications requiring aggressive critical care. Due to the rarity of this class of tumors, the survival of critically ill sarcoma patients has not been well studied.

The treatment of such patients requires a multidisciplinary approach, with coordination among oncologists, critical care physicians, consulting services, ancillary staff, and patients’ families. Despite the availability of advanced life support devices in intensive care centers in the United States, it is difficult for both patients and physicians to objectively determine the effect of such heroic measures on prognosis or quality of life. Studies up to the 1990s had shown that among all diseases, patients with cancer had the lowest intensive care unit (ICU) survival rates, and the majority of these patients died soon after their hospital discharge [3–5]. However, over the past two decades, the approach to treatment in such cases has been shifting. Some studies have shown that patients lacking predictors of poor survival outcomes are considered as good candidates for aggressive therapy [6–8].

The primary objective of our study was to determine the ICU and post-ICU survival rates of critically ill sarcoma patients. Secondary objectives included determining the modifiable and nonmodifiable predictors of poor survival.

## Methods

### Patient population

After obtaining institutional review board approval, we retrospectively reviewed the electronic medical records of 212 critically ill patients with sarcoma who had been admitted to the ICU at The University of Texas MD Anderson Cancer Center between January 1, 2006, and December 31, 2012. For the purpose of this analysis, we limited the study population to patient’s first ICU admission only. We also excluded from the study population patients who had been admitted to the ICU for perioperative care.

### Study design

This retrospective study was designed to identify predictors of poor survival that contribute to ICU mortality. ICU mortality was defined as the percentage of patients with sarcoma who died in the ICU among total number of patients admitted to the ICU with sarcoma during the study period. Secondary outcomes were in-hospital mortality and 1, 2, and 6-month survival rates, which were defined by similar means. Overall survival (OS) for each patient was measured from the time of initial ICU admission to the last date of contact or death. We defined *ICU survival* as short-term or acute survival and 6-*month survival* as long-term survival. Median follow-up time was calculated from the date of ICU admission.

Patient’s current and previous chemotherapy regimens were examined to evaluate the impact of various chemotherapy treatments on survival. Tumor burden was noted by recording the various sites of metastasis, such as head and neck, musculoskeletal, heart, lung, liver, gastrointestinal tract, and spleen. The Charlson comorbidities index (CCI) was used to assess the role of serious comorbid disease in the survival of patients in the study [9].

Clinical findings present at the time of ICU admission were recorded to assess acute illness (definitions in parentheses). Variables recorded included acute renal failure, anemia, thrombocytopenia, pancytopenia, hemorrhage (including bleeding from gastrointestinal tract), lactic acidosis, heart failure, pulmonary embolism, respiratory failure (use of noninvasive or invasive mechanical ventilation), cardiac arrest, atrial fibrillation, cardiac dysrhythmia, pneumonia, septic shock, hypotension (systolic blood pressure <100 or on vasopressors), hypertension, neutropenic fever (absolute neutrophil count <1500), altered mental status, and malnutrition (patient on feeding tube or total parental nutrition).

Severity of illness at the time of ICU admission was measured by Sequential Organ Failure Assessment (SOFA) scores. Maximum SOFA scores and discharge SOFA scores were also obtained to track the patients’ progress while they were in the ICU. Change in SOFA scores were calculated by subtracting the admission SOFA scores from the maximum SOFA scores. Organ failure at the time of ICU admission was determined by an admission SOFA score ≥2 per organ system. The total number of organ failures was calculated for each patient.

### Analysis

Patient characteristics were tabulated and compared between groups by using the Chi square test or Fisher exact test as appropriate for categorical variables and by the nonparametric Wilcoxon rank sum test for continuous variables. A multivariate logistic regression model was fitted to examine the relationship between death in the ICU and clinical characteristics. Patients who were lost to follow-up or alive were censored at their dates of last contact. The Kaplan–Meier product limit method was used to estimate the survival outcomes of all patients by groups; the log-rank statistic was used to compare groups. Cox proportional hazards models were fitted to determine the association of patient and clinical characteristics with OS. Variables that had significant univariate log-rank p values were candidates for the multivariate model. Results were expressed in hazard ratios (HRs), odds ratios (ORs) and 95 % confidence intervals (CIs). p values of less than 0.05 were considered statistically significant; all tests were two-sided. Statistical analyses were carried out by using SAS 9.4 (SAS Institute Inc., Cary, NC) and S-Plus 8.2 (TIBCO Software Inc, Palo Alto, CA, USA).

## Results

### Patient characteristics

We identified a total of 212 sarcoma admissions to the ICU at MD Anderson between January 1, 2005, and December 31, 2012. We excluded 23 ICU admissions of patients who were admitted to the ICU multiple times during the study course. Of the remaining 189 admissions, 17 perioperative admissions were excluded, leaving a sample of 172 first-time ICU admissions. The study population was 45.9 % male with median age of 52 years (interquartile range [IQR] 38–62 years) (Supplemental Digital Content—Table 1). The most common sarcoma subgroups were unclassified high-grade sarcoma (25 %), bone sarcoma (Ewing sarcoma, osteosarcoma, and chondrosarcoma; 17.4 %), vascular sarcoma (angiosarcoma and epithelioid hemangioendothelioma; 9.9 %), and leiomyosarcoma (7.6 %). The ICU mortality rate was 23.3 % (95 % CI 16.9–29.6 %), and the hospital mortality rate was 29.7 % (95 % CI 22.9–37.1 %). The median Charlson comorbidity index was 6 (IQR 6–7) owing to presence of metastatic cancer in most of the patients at the time of admission.

**Table 1:** Patient and clinical characteristics by Alive or Death at the Intensive Care Unit (ICU)

	All patients^a^ (N = 172)	Alive at ICU discharge^b^ (N = 132)	Death at ICU^b^ (N = 40)	p*
Age (y)				
Median (IQR)	52 (38–62)	52 (38–62)	53.5(36–66)	0.65
Gender				
Female	93 (54.1 %)	71 (76.3 %)	22 (23.7 %)	
Male	79 (45.9 %)	61 (77.2 %)	18 (22.8 %)	0.89^†^
Histological diagnosis				
Unclassified high-grade sarcoma	43 (25.0 %)	36 (83.7 %)	7 (16.3 %)	
Bone sarcoma^c^	30 (17.4 %)	21 (70 %)	9 (30 %)	
Vascular^d^	17 (9.9 %)	16 (94.1 %)	1 (5.9 %)	
GIST^e^	11 (6.4 %)	10 (90.9 %)	1 (9.1 %)	
MFH^e^	11 (6.4 %)	6 (54.5 %)	5 (45.5 %)	
Muscle	10 (5.8 %)	7 (70 %)	3 (30 %)	
Leiomyosarcoma	13 (7.6 %)	12 (92.3 %)	1 (7.7 %)	
Liposarcoma	7 (4.1 %)	4 (57.1 %)	3 (42.9 %)	
Synovial sarcoma	9 (5.2 %)	5 (55.6 %)	4 (44.4 %)	
Others	21 (12.2 %)	15 (71.4 %)	6 (28.6 %)	0.09
Status of malignancy				
First course of chemotherapy	29 (16.9 %)	20 (69 %)	9 (31 %)	
Progression	59 (34.3 %)	45 (76.3 %)	14 (23.7 %)	
Stable disease or partial remission	61 (35.5 %)	50 (82 %)	11 (18 %)	
Complete remission	4 (2.3 %)	4 (100 %)	0 (0 %)	
Mixed response	6 (3.5 %)	4 (66.7 %)	2 (33.3 %)	
Unknown	13 (7.6 %)	9 (69.2 %)	4 (30.8 %)	0.57
Site of malignancy				
Head and neck	15 (8.7 %)	11 (73.3 %)	4 (26.7 %)	
Thoracic	43 (25 %)	34 (79.1 %)	9 (20.9 %)	
Abdomen	71 (41.3 %)	55 (77.5 %)	16 (22.5 %)	
Extremities	43 (25 %)	32 (74.4 %)	11 (25.6 %)	0.94
Organ metastasis				
Lung				
No	86 (50 %)	69 (80.2 %)	17 (19.8 %)	
Yes	86 (50 %)	63 (73.3 %)	23 (26.7 %)	0.28
Liver				
No	142 (82.6 %)	108 (76.1 %)	34 (23.9 %)	
Yes	30 (17.4 %)	24 (80 %)	6 (20 %)	0.64
Other				
No	79 (45.9 %)	57 (72.2 %)	22 (27.8 %)	
Yes	93 (54.1 %)	75 (80.6 %)	18 (19.4 %)	0.19
Number of organ metastasis				
0	42 (24.4 %)	32 (76.2 %)	10 (23.8 %)	
1	54 (31.4 %)	39 (72.2 %)	15 (27.8 %)	
≥2	76 (44.2 %)	61 (80.3 %)	15 (19.7 %)	0.56
Treatment				
Current chemotherapy regimen				
None	43 (25 %)	34 (79.1 %)	9 (20.9 %)	
Adriamycin-based	68 (39.5 %)	50 (73.5 %)	18 (26.5 %)	
Gemcitabine-based	20 (11.6 %)	13 (65 %)	7 (35 %)	
Targeted therapy	41 (23.8 %)	35 (85.4 %)	6 (14.6 %)	0.29
No. cycles of current chemotherapy, median (IQR^e^)	1 (0–3)	1 (0–3)	1 (1–2)	0.80^†^
No. cycles of prior chemotherapies, median (IQR)	1 (0–3)	1 (0–3)	0 (0–2)	0.037^†^
No. cycles of prior chemotherapies				
0–1	114 (66.3 %)	88 (77.2 %)	26 (22.8 %)	
≥2	58 (33.7 %)	44 (75.9 %)	14 (24.1 %)	0.85
Radiation				
No	109 (63.7 %)	80 (73.4 %)	29 (26.6 %)	
Yes	62 (36.3 %)	51 (82.3 %)	11 (17.7 %)	0.19
Clinical conditions present at ICU admission				
Anemia	144 (83.7 %)	110 (76.4 %)	34 (23.6 %)	0.80
Hypotension	92 (53.5 %)	63 (68.5 %)	29 (31.5 %)	0.006
Septic shock	53 (30.8 %)	33 (62.3 %)	20 (37.7 %)	0.003
Bacteremia	21 (12.2 %)	14 (66.7 %)	7 (33.3 %)	0.24
Thrombocytopenia	86 (5 %)	68 (79.1 %)	18 (20.9 %)	0.47
Respiratory failure	74 (43 %)	39 (52.7 %)	35 (47.3 %)	<0.0001
Acidosis	70 (40.7 %)	42 (60 %)	28 (40 %)	<0.0001
Altered mental status	65 (37 %)	42 (64.6 %)	23 (35.4 %)	0.003
Abnormal glucose	60 (34.9 %)	42 (70 %)	18 (30 %)	0.13
Acute renal failure	58 (33.7 %)	35 (60.3 %)	23 (39.7 %)	0.0003
Pancytopenia	58 (33.7 %)	47 (81 %)	11 (19 %)	0.34
Pneumonia	51 (29.7 %)	32 (62.7 %)	19 (37.3 %)	0.005
Neutropenia (ANC^e^ <1500/mm^3^)	51 (29.7 %)	40 (78.4 %)	11 (21.6 %)	0.73
Cardiac dysrhythmia	35 (20.3 %)	23 (65.7 %)	12 (34.3 %)	0.08
Heart failure	32 (18.6 %)	21 (65.6 %)	11 (34.4 %)	0.10
Hypertension	25 (14.5 %)	22 (88 %)	3 (12 %)	0.20*
Malnutrition (protein/calorie) NOS	23 (13.4 %)	15 (65.2 %)	8 (34.8 %)	0.16
Hemorrhage	15 (8.7 %)	12 (80 %)	3 (20 %)	1.0*
Gastrointestinal hemorrhage	13 (7.6 %)	12 (92.3 %)	1 (7.7 %)	0.30*
Cardiac arrest	11 (6.4 %)	3 (27.3 %)	8 (72.7 %)	0.0004*
Pulmonary embolism	11 (6.4 %)	8 (72.7 %)	3 (27.3 %)	0.72*
Atrial fibrillation	10 (5.8 %)	7 (70 %)	3 (30 %)	0.70*
Seizures/convulsions	9 (5.2 %)	8 (88.9 %)	1 (11.1 %)	0.69*
ICU admission data				
Mechanical v entilator	75 (43·6 %)	40 (30.3 %)	35 (87.5 %)	<0.0001
Charlson comorbidity index, median (IQR)	6 (6–7)	6 (6–7)	6 (4.5–7)	0.70^†^
≤2	26 (15.1 %)	19 (73.1 %)	7 (26.9 %)	
>2	146 (84.9 %)	113 (77.4 %)	33 (22.6 %)	0.63
SOFA admission score, median (IQR)	7 (4–10)	6 (3.5–9)	10 (6.5–14)	<0.0001^†^
Max SOFA admission score, median (IQR)	8 (5–12)	7 (4–9.5)	14 (10–17)	<0.0001^†^
SOFA discharge score, median (IQR)	5 (3–8)	4 (2–6)	10 (7.5–13.5)	<0.0001^†^
No. organ failures, median (IQR)	1 (0–2)	1 (0–2)	2 (1–3)	<0.0001

* Fisher exact p value

^†^Wilcoxon rank-sum test

^a^Count (column %—percent of admissions with that variable) are presented unless specified

^b^Count (row  %—percent of N in column with All patients) are presented unless specified

^c^Ewing sarcoma, osteosarcoma, chondrosarcoma

^d^Angiosarcoma, epithelioid hemangioendothelioma

^e^Gastrointestinal stromal tumor, Malignant fibrous histiocytoma, Interquartile range, Sequential Organ Failure Assessment, Absolute Neutrophil Count

### Death in the ICU

There were 40 patient deaths (23.3 %) in the ICU. In the univariate logistic regression model for the death at the ICU, short-term mortality did not correlate with tumor site, histology, disease status, presence of metastatic disease, Charlson comorbidity index or neutropenia at the time of ICU admission. However, patients with clinical findings of acidosis, acute renal failure, cardiac arrest, hypotension (including septic shock), pneumonia, septic shock or respiratory failure at the time of ICU admission had worse outcomes than patients who lacked these findings on admission (Supplemental Digital Content—Tables 1, 2).

**Table 2:** Univariate logistic regression model for death at ICU

	Odds Ratio	95 % CI		p
Reason for ICU admission acidosis: yes v. no	5.000	2.317	10.788	<0.0001
Reason for ICU admission renal failure: yes v. no	3.750	1.795	7.831	0.0004
Reason for ICU admission cardiac arrest: yes v. no	10.750	2.699	42.824	0.0008
Reason for ICU admission cardiac dysrhythmia: yes v. no	2.031	0.902	4.575	0.0873
Reason for ICU admission hypotension: yes v. no	2.887	1.332	6.258	0.0072
Reason for ICU admission pneumonia: yes v. no	2.827	1.353	5.910	0.0057
Reason for ICU admission respiratory failure: yes v. no	16.691	6.086	45.775	<0.0001
Reason for ICU admission septic shock: yes v. no	3.000	1.439	6.253	0.0034
Number of organ failures	1.933	1.417	2.637	<0.0001
Number of mets	0.997	0.745	1.334	0.9835
Histology diagnosis				
Unclassified high grade sarcoma	1.000			
Bone (Ewing, Osteo, Chondrosarcoma)	2.204	0.716	6.788	0.1685
Vascular	0.321	0.036	2.833	0.3068
GIST	0.514	0.056	4.685	0.5552
MFH	4.286	1.019	18.029	0.0471
Muscle	2.204	0.456	10.661	0.3258
Leiomyosarcoma	0.429	0.048	3.848	0.4493
Liposarcoma	3.857	0.703	21.153	0.1200
Synovial sarcoma	4.114	0.878	19.270	0.0726
Others	2.057	0.592	7.149	0.2564

The median SOFA scores on admission were 6 (IQR 3.5–9) in ICU survivors and 10 (IQR 6.5–14) in non-survivors. In addition, the median maximum SOFA scores were 7 (IQR 4–9.5) in survivors and 14 (IQR 10–17) in non-survivors, and the median discharge SOFA scores were 4 (IQR 2–6) in survivors and 10 (IQR, 7.5–13.5) in non-survivors. Patients with admission SOFA scores of 11 or more had a lower ICU survival rate than did those with scores less than 11 (45.7 vs 80 % or more) (Table 2). An increase of 6 or more in the SOFA score from the time of admission significantly affected short- (ICU survival 13.3 %) and long-term outcomes (OS 6.7 %) (Table 3).

**Table 3:** Trends in Sequential Organ Failure Assessment (SOFA) scores and their impact on ICU and overall survival

Increase in SOFA score during ICU stay (Maximum SOFA- Admission SOFA)	No. patients in subgroup	Patients surviving ICU stay, %	Patients surviving till end of study, %	Median survival, d (range)
SOFA scores during ICU stay				
0 or less	103	86.4	22.3	187 (108–347)
1–3	43	81.4	27.9	96 (21–193)
4–5	10	60.0	10.0	12 (2–85)
≥6	15	13.3	6.7	17 (6–22)
Admission SOFA scores				
0–4	50	88.0	26.0	159 (49–289)
5–7	46	84.8	21.7	182 (38–347)
8–10	41	80.5	24.4	185 (58–494)
≥11	35	45.7	11.4	6 (2–19)
Change in SOFA scores of 35 patients with admission SOFA scores of 11 or more				
≤0	18	44.4	5.6	4.5 (0.5–9)
1	6	83.3	33.3	56 (4–NE)
2	3	66.7	33.3	13 (2–NE)
≥3	8	12.5	0.0	6 (1–46)

*ADM SOFA* SOFA score at the time of ICU admission, *MAXOFA* maximum SOFA score, *NE* not estimated

Multivariate logistic regression model for death in the ICU showed that SOFA admission score (OR 1.23, 95 % CI 1.12–1.35, p < 0.0001) was associated with death in the ICU. Variables that were initially included in the model and then reduced in a stepwise selection were SOFA admission score and number of metastatic sites.

### OS from ICU admission

**Fig. 1:**
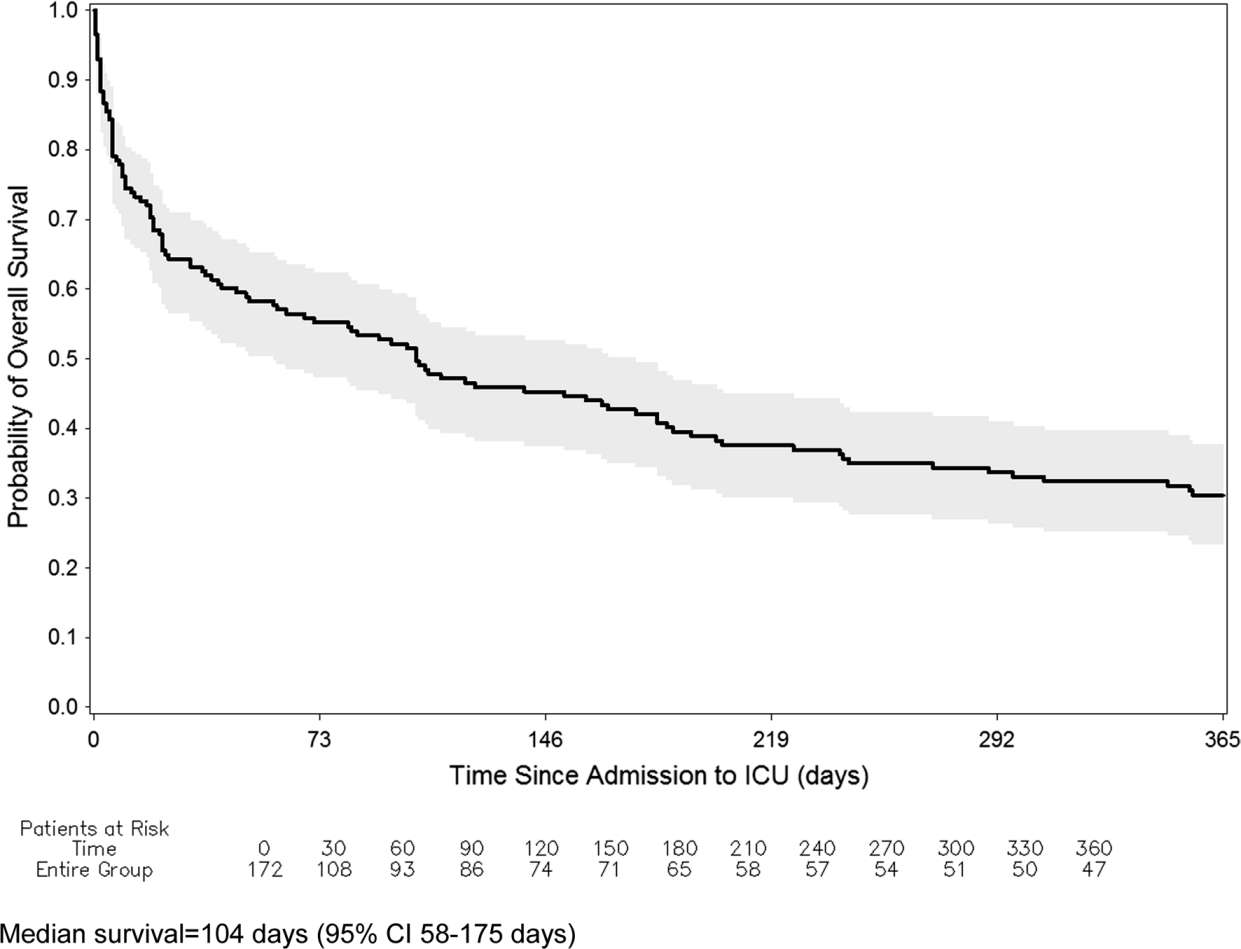
Kaplan-Meier estimates of overall survival

Median follow-up among all patients was 2.9 months (range 0.02–77.5 months). At the time of this analysis, 135 patients (78.5 %) had died. For the whole cohort, 1, 2, and 6-month OS rates were 64, 57, and 41 %, respectively (Table 4). The Kaplan–Meier curves for OS are shown in Fig. 1.

**Table 4:** Overall survival estimates by patients and clinical characteristics in percentages

	No of patients	No of deaths	1-month overall survival estimate (95 % CI)	2-month overall survival estimate (95 % CI)	6-month overall survival estimate (95 % CI)	p
All patients	172	135	64 (57–71)	57 (49–64)	41 (33–48)	
Age (years)						
<65	139	109	64 (56–72)	56 (47–64)	41 (33–50)	
≥65	33	26	64 (45–77)	61 (42–75)	37 (21–54)	0.91
Gender						
Female	93	72	66 (56–75)	60 (49–69)	45 (34–55)	
Male	79	63	62 (50–71)	48 (37–59)	36 (25–47)	0.33
Histological diagnosis						
Unclassified high-grade sarcoma	43	34	67 (51–79)	58 (42–71)	46 (30–60)	
Bones^a^	30	25	70 (50–83)	60 (40–75)	38 (21–55)	
Vascular^b^	17	16	64 (36–82)	51 (25–72)	32 (12–54)	
GIST	11	7	73 (37–90)	64 (30–85)	45 (17–71)	
MFH	11	8	55 (23–78)	55 (23–78)	36 (11–63)	
Muscle	10	9	60 (25–83)	60 (25–83)	20 (3–47)	
Leiomyosarcoma	13	7	84 (50–96)	84 (50–96)	75 (40–91)	
Liposarcoma	7	7	29 (4–61)	29 (4–61)	29 (4–61)	
Synovial sarcoma	9	6	56 (20–80)	56 (20–80)	56 (20–80)	
Others	21	16	56 (33–74)	45 (23–65)	28 (11–49)	0.07
Status of malignancy						
First course of chemotherapy	29	22	66 (45–8)	55 (35–71)	41 (22–59)	
Progression	59	56	49 (36–61)	37 (25–49)	17 (9–27)	
Stable disease or partial remission	61	41	75 (62–84)	72 (58–81)	61 (47–72)	
Complete remission	4	2	100	100	75 (13–96)	
Mixed response	6	5	67 (19–90)	67 (19–90)	67 (19–90)	
Unknown	13	9	68 (36–87)	68 (36–87)	29 (7–56)	<0.0001
Site of malignancy						
Head and neck	15	11	52 (25–73)	52 (25–73)	29 (9–53)	
Thoracic	43	33	67 (51–79)	60 (43–73)	46 (30–60)	
Abdomen	71	57	62 (49–72)	53 (41–64)	37 (26–48)	
Extremities	43	34	7 (54–81)	63 (47–75)	46 (31–60)	0.86
Organ metastasis						
Lung						
No	86	62	66 (55–75)	59 (48–69)	46 (35–56)	
Yes	86	73	62 (51–72)	55 (43–65)	36 (25–46)	0.02
Liver						
No	142	109	66 (58–73)	59 (51–67)	46 (37–54)	
Yes	30	26	56 (36–72)	45 (27–62)	17 (6–33)	0.0093
Other						
No	79	55	66 (54–75)	63 (51–73)	49 (38–60)	
Yes	93	80	63 (52–72)	52 (41–62)	33 (24–43)	0.0091
Number of organ metastases						
0	42	25	71 (55–83)	69 (53–81)	59 (43–72)	
1	54	43	59 (45–71)	53 (39–65)	40 (26–53)	
2+	76	67	64 (52–74)	53 (41–64)	31 (21–42)	0.001
Localized disease						
No	130	110	62 (53–7)	53 (44–61)	34 (26–43)	
Yes	42	25	71 (55–83)	69 (53–81)	59 (43–72)	<0.0001
Treatment						
Current chemotherapy regimen						
None	43	31	57 (41–7)	52 (36–66)	39 (24–53)	
Adriamycin-based chemotherapy	68	51	69 (56–78)	66 (53–76)	51 (38–63)	
Gemcitabine-based therapy	20	18	5 (27–69)	45 (23–65)	25 (9–45)	
Targeted therapy	41	35	71 (54–82)	54 (37–67)	34 (20–49)	0.31
Radiation						
No	109	85	63 (54–72)	59 (49–67)	39 (29–48)	
Yes	62	50	65 (52–76)	53 (40–65)	43 (30–55)	0.86
Clinical conditions present at ICU admission						
Anemia	144	113	66 (57–73)	59 (50–66)	43 (35–51)	0.60
Hypotension	92	73	57 (46–66)	51 (40–61)	42 (32–52)	0.96
Septic shock	53	44	49 (35–62)	45 (32–58)	33 (21–46)	0.10
Bacteremia	21	17	57 (34–75)	57 (34–75)	43 (22–62)	0.58
Thrombocytopenia	86	63	71 (60–79)	64 (52–73)	49 (37–59)	0.037
Respiratory failure	74	62	46 (34–56)	41 (30–52)	26 (16–37)	<0.0001
Acidosis	70	58	47 (35–58)	41 (30–53)	35 (24–46)	0.011
Altered mental status	65	57	52 (39–63)	42 (30–54)	25 (15–36)	0.001
Abnormal glucose	60	50	60 (46–71)	49 (36–61)	25 (14–37)	0.017
Acute renal failure	58	49	48 (35–60)	40 (27–52)	29 (18–41)	0.016
Pancytopenia	58	41	79 (66–88)	74 (61–83)	57 (43–69)	0.006
Respiratory abnormality	53	38	73 (59–83)	64 (49–75)	49 (35–62)	0.036
Pneumonia	51	42	53 (38–65)	49 (35–62)	35 (22–48)	0.41
Neutropenia (ANC <1500/mm^3^)	51	36	78 (64–87)	70 (56–81)	58 (43–70)	0.013
Cardiac dysrhythmia	35	26	57 (39–72)	51 (34–66)	46 (29–61)	0.96
Heart failure	32	25	63 (44–77)	56 (37–71)	53 (34–68)	0.80
Hypertension	25	15	80 (58–91)	71 (48–85)	61 (39–78)	0.049
Malnutrition (protein/calorie) NOS	23	21	60 (37–77)	60 (37–77)	28 (11–47)	0.047
Hemorrhage (non–gastrointestinal)	15	9	64 (34–83)	64 (34–83)	47 (19–71)	0.44
Other pulmonary insufficiency	14	11	71 (41–88)	64 (34–83)	64 (34–83)	0.71
Gastrointestinal hemorrhage	13	9	62 (31–82)	54 (25–76)	38 (14–63)	0.51
Cardiac arrest	11	8	27 (7–54)	27 (7–54)	27 (7–54)	0.17
Pulmonary embolism	11	10	73 (37–90)	63 (29–84)	32 (8–59)	0.21
Atrial fibrillation	10	7	70 (33–89)	70 (33–89)	6 (25–83)	0.40
Seizures	9	8	67 (28–88)	44 (14–72)	30 (5–61)	0.54
ICU admission data						
Charlson comorbidity index						
≤2	26	15	69 (48–83)	69 (48–83)	54 (33–71)	
>2	146	120	63 (55–71)	55 (46–62)	38 (30–46)	0.0031
Charlson comorbidity index						
<6	42	25	71 (55–83)	69 (53–81)	59 (43–72)	
≥6	130	110	62 (53–70)	53 (44–61)	34 (26–43)	<0.0001
SOFA maximum score						
<8	79	60	82 (72–89)	71 (60–80)	49 (38–60)	
≥8	93	75	49 (39–59)	45 (34–55)	33 (24–43)	0.005
SOFA discharge score						
<5	77	59	80 (69–88)	71 (59–80)	47 (35–58)	
≥5	95	76	51 (41–61)	46 (36–56)	36 (26–45)	0.12
No. organ failures						
1	59	47	76 (63–85)	69 (56–79)	53 (40–65)	
≥2	113	88	58 (48–66)	50 (41–59)	34 (25–43)	0.12

*ADM SOFA* SOFA score at the time of ICU admission, *MAXOFA* maximum SOFA score, *NE* not estimated

^a^Ewing sarcoma, osteosarcoma, chondrosarcoma

^b^Angiosarcoma, epithelioid hemangioendothelioma

In multivariable Cox proportional hazards models (Table 5), patients with gastrointestinal stromal tumors (HR 0.281, 95 % CI 0.119–0.662, p = 0.004) and leiomyosarcoma (HR 0.375, 95 % CI 0.160–0.880, p = 0.02) had a lower risk of death than did patients with unclassified high-grade sarcoma. Patients with higher Charlson Comorbidity Index (HR 1.139, 95 % CI 1.023–1.268, p = 0.02) and those with higher SOFA scores at discharge (HR 1.210, 95 % CI 1.141–1.283, p < 0.0001) also had higher risk of death (Table 5). Median survival rates were lower in patients with acute renal failure, cardiac arrest, pneumonia, septic shock, and respiratory failure.

**Table 5:** Multivariable cox proportional hazards model for overall survival

Variable	Hazard ratio	95 % CI P		p
Histological diagnosis				0.006
Unclassified high-grade sarcoma	1.000			
Bones^a^	0.881	0.516	1.506	0.64
Vascular^b^	1.657	0.862	3.185	0.13
GIST	0.281	0.119	0.662	0.004
MFH	0.674	0.236	1.929	0.46
Muscle	2.048	0.917	4.573	0.08
Leiomyosarcoma	0.375	0.160	0.880	0.02
Liposarcoma	1.412	0.575	3.468	0.45
Synovial sarcoma	0.793	0.293	2.143	0.65
Others	0.746	0.373	1.493	0.41
Status of malignancy				0.003
Complete remission	1.000			
First course of chemotherapy	2.323	0.473	11.425	0.30
Mixed response	2.720	0.444	16.677	0.28
Progression	5.172	1.075	24.874	0.04
Stable disease	2.262	0.501	10.218	0.29
Reason for ICU admission pancytopenia				
No				
Yes	0.351	0.226	0.546	<0.0001
Reason for ICU admission respiratory failure				
No				
Yes	1.703	1.125	2.579	01
CCI (continuous)	1.139	1.023	1.268	02
SOFA score at discharge (continuous)	1.210	1.141	1.283	<0.0001

Initially included in the model and then reduced by stepwise selection method: histology diagnosis, status of malignancy, number of organ metastasis, number of organ failures, and reason for ICU admission: acidosis, acute renal failure, pancytopenia, respiratory failure, and septic shock. There were 13 patients without known malignancy status who were not included in this analysis

*CI* confidence interval, *GIST* Gastrointestinal stromal tumor, *MFH* Malignant fibrous histiocytoma,* CCI* Charlson comorbidities index,* SOFA* Sequential Organ Failure Assessment

^a^Ewing sarcoma, osteosarcoma, chondrosarcoma

^b^Angiosarcoma, epithelioid hemangioendothelioma

## Discussion

Decisions about the intensive care treatment of critically ill cancer patients with poor prognoses are challenging and need to be evaluated on a patient-by-patient basis. Our study benefits both physicians and family members by providing objective data on ICU mortality, long-term survival, and objective predictors of survival. Our results showed that among sarcoma patients, the ICU mortality, in-hospital mortality, and long-term survival rates were 23.3, 29.7, and 41 %, respectively. Patients who were admitted due to acute renal failure, cardiac arrest, septic shock, or respiratory failure had poor ICU outcomes and median survival durations ranging from 1 to 21 days. The median SOFA scores at the times of admission and discharge were significantly lower in ICU survivors than in non-survivors. An increase in SOFA score during the ICU stay is an important predictor of poor survival outcomes. We also determined that a higher number of organ failures was associated with an increased risk of ICU mortality (Supplemental Digital Content—Table 1).

The ICU mortality rates observed in the study population were consistent with those in the existing medical literature and promote a case for a higher level of care with aggressive monitoring in patients who lack predictors of poor outcomes. Our data have shown that adequate cardiac, renal, and respiratory functions play a key role in acute survival. Serial SOFA scores may serve as an objective measure for short-term and long-term prognosis for sarcoma. There appears to be significant heterogeneity in ICU mortality among all cancer patients because of a lack of data comparing similar histologies. Our patient population had an ICU mortality rate of 23 %, which is comparable to the 20 % mortality in solid tumors [10]. The same study reported that ICU and in-hospital mortality rates for cancer patients were not significantly different from those of non-cancer patients [10]. However, among patients with multiple organ failures; mortality rates were higher in cancer patients than in non-cancer patients. This finding is consistent with our study, in which the number of organ failures was directly proportional to ICU mortality. Among mortality statistics reported for specific tumors, patients with head and neck cancer, lung cancer, and gynecological malignancies have been shown to have ICU mortality rates of 39, 36, and 17.3 %, respectively [11–13]. In contrast, patients with hematological cancers have been reported to have higher ICU mortality rates: 48.3 and 56 % in two separate studies [14, 15]. These higher rates may be attributed to increased severity of illness and higher incidence of sepsis due to associated leukopenia in these patients [10].

Several ICU scores; including MODS, APACHE II, SAPS II, and SOFA, have been used as objective ways to describe mild to severe organ dysfunction [16–19]. In our study, we used SOFA scores, which have been calibrated to predict ICU and in-hospital mortality rates in cancer patients by evaluating the combination of clinical conditions that lead to ICU admission [20–23].

We found out those patients who had high SOFA scores on admission did worse than patients with lower SOFA scores. Another important objective finding was that patients whose SOFA scores continued to rise following admission to the ICU had the worst outcomes, making these patients appropriate candidates for early supportive and palliative care. As a general guide, increase in scores of 6 or more since admission led to drastic changes in outcome and ICU survival decreased from 60 to 13.3 % (Table 3). A patient who is admitted with a SOFA score of 11 or higher and their score increases by 3 or more has dismal ICU survival of 12.5 %. Such a patient should be considered for transition to hospice (Table 3).

Among individual clinical findings on ICU admissions, patients with cardiac arrest had the worst prognosis, with an ICU mortality rate of 72 % (8/11) and a median OS of 24 h. However, cardiopulmonary resuscitation has been shown to be a non-beneficial intervention in more than 90 % of the patients with cancer [24–26]. In our univariate analysis, patients with respiratory failure had worse outcomes than patients with good respiratory status. Mechanical ventilation has been shown to be associated with increased mortality rates (73 %) in cancer patients, especially those with disseminated disease and poor performance status at the time of ICU admission [27]. Similar findings have been observed in patients with hematological, lung, and head and neck cancers in the ICU setting (64–74 %) [20, 28–30].

Hypotension and septic shock are additional clinical findings that correlated with higher ICU mortality in our multivariate analysis. Sepsis is one of the leading causes of ICU admission in cancer patients. However, patients with infections leading to shock and vasopressor use tend to do poorly and should be managed conservatively [10, 13, 15, 27]. Patients with acute kidney injury also have high acute mortality. This trend is more commonly seen in patients with hematological malignancies [31]. Furthermore, renal replacement therapy has been associated with high ICU mortality [24]. Given their bleak prognoses, patients with these individual or combinations of clinical findings should be triaged early so that inappropriate use of aggressive therapy can be avoided.

There are limited data evaluating long-term survival rates of critically ill cancer patients after their discharge from the ICU. Six-month OS in sarcoma patients was 41 % in our study, which is comparable to the 6-month mortality rates of 59.3 and 66 % reported in critically ill patients with hematological malignancies [28, 30]. However, long-term mortality rates of 63 to 98 % have been reported in patients with lung cancer. The major predictors of long-term prognosis in these patients were dependence on mechanical ventilation during the ICU stay and progression of cancer after discharge from the ICU [11]. In our study, we found that the status of malignancy, metastatic disease, Charlson comorbidity index and SOFA scores at discharge, and the presence of respiratory failure or cytopenia at the time of ICU admission were significant predictors of survival outcomes in both univariate and multivariate analyses (Table 4). Therefore, when looking at patterns of long-term survival after ICU stay, the above-mentioned clinical prognostic factors should be considered early in the clinical course.

There were a number of limitations to this study. First, this is a single-institution retrospective study covering a limited number of years. We are a tertiary care referral center for sarcomas, so the results of our study may not be applicable to smaller hospitals or low-volume centers. Each hospital has its own ICU admission and discharge policies which may bias the results of a single center study. Second, our study did not have a control group to compare the outcomes of critically ill sarcoma patients who were not admitted to the ICU and were managed conservatively. Even though it is a large collection of a rare tumor our study outcomes were limited to only 40 events in the ICU. Due to a small number of events and multiple risk factors, it is difficult to make a generalized presumption of which risk factor individually impacted the short term survival. In addition, quality-of-life measures and performance status scores could not be reliably collected in the retrospective setting.

In conclusion, our study is the largest study to date of OS in sarcoma patients admitted to the ICU. In our study span of 7 years, we have shown that OS in sarcoma is comparable with that of critically ill patients with other solid tumors. The admission SOFA scores and change in the SOFA scores during ICU stay are highly reliable indicators of probability of survival and should be used in decision making in critically ill patients. Our results suggest that patients with advanced malignancy that are admitted to the ICU for respiratory failure, cardiac arrest, septic shock, acute renal failure or acidosis and have high SOFA score with subsequent worsening in the ICU have very poor prognosis. Based on the retrospective data which needs further validation we can recommend that judicious approach should be taken in patients with predictors of poor survival before subjecting them to aggressive treatment.
